# Experimental Analyses and Predictive Modelling of Ultrasonic Welding Parameters for Enhancing Smart Textile Fabrication

**DOI:** 10.3390/s24051488

**Published:** 2024-02-25

**Authors:** Mohamed Baraya, Mohamed S. El-Asfoury, Omnia O. Fadel, Ahmed Abass

**Affiliations:** 1 Department of Production Engineering and Mechanical Design, Faculty of Engineering, Port Said University, Port Fuad 42526, Egypt; mohamed.baraya@eng.psu.edu.eg (M.B.); mohamed.saad@eng.psu.edu.eg (M.S.E.-A.); omniaothman@eng.psu.edu.eg (O.O.F.); 2 Department of Materials, Design and Manufacturing Engineering, School of Engineering, University of Liverpool, Liverpool L69 3GH, UK

**Keywords:** ultrasonic welding, smart textiles, copper wire joining, artificial neural network (ANN), full factorial experimental design (FFD)

## Abstract

This study aims to illustrate the design, fabrication, and optimisation of an ultrasonic welding (UW) machine to join copper wires with non-woven PVC textiles as smart textiles. The study explicitly evaluates UW parameters’ impact on heat generation, joint strength, and electrical properties, with a comprehensive understanding of the process dynamics and developing a predictive model applicable to smart textiles. The methodological approach involved designing and manufacturing an ultrasonic piezoelectric transducer using ABAQUS finite element analyses (FEA) software and constructing a UW machine for the current purpose. The full factorial design (FFD) approach was employed in experiments to systematically assess the influence of welding time, welding pressure, and copper wire diameter on the produced joints. Experimental data were meticulously collected, and a backpropagation neural network (BPNN) model was constructed based on the analysis of these results. The results of the experimental investigation provided valuable insights into the UW process, elucidating the intricate relationship between welding parameters and heat generation, joint strength, and post-welding electrical properties of the copper wires. This dataset served as the basis for developing a neural network model, showcasing a high level of accuracy in predicting welding outcomes compared to the FFD model. The neural network model provides a valuable tool for controlling and optimising the UW process in the realm of smart textile production.

## 1. Introduction

Smart textiles, also known as e-textiles or smart fabrics, are a transformative category of materials with integrated electronic components that can sense, respond to, and communicate data [1]. These textiles have gained increasing importance in various fields due to their versatility and potential applications [2]. Smart textiles offer many benefits, from enhancing our daily lives to advancing industries and healthcare [1]. Their applications encompass wearables that monitor health and fitness, such as heart rate or temperature, and offer real-time feedback. In the fashion industry, they enable interactive clothing and accessories that change colour or pattern in response to environmental stimuli. Moreover, the military and sports industries are adopting smart textiles for improved performance and safety [3]. In healthcare, smart textiles assist in remote patient monitoring and developing prosthetics. Furthermore, the automotive and aerospace sectors utilise these materials to enhance passenger comfort and safety [4]. Smart textiles are also crucial in the Internet of Things (IoT), facilitating seamless data collection, transmission, and processing in smart homes, agriculture, and industrial automation [5].

Smart textiles are created through various fabrication methods, each with unique advantages and disadvantages. Traditional methods include embroidery and weaving, where conductive threads are integrated into the textile structure [6,7]. Another method involves screen printing, where conductive ink is applied to fabric to create conductive patterns [8,9]. Emerging techniques include three-dimensional (3D) knitting and additive manufacturing, which enable intricate designs and precise integration of electronic components [10,11]. Embroidery and weaving provide durability and flexibility but may lack precision [12]. Screen printing offers cost-effectiveness and scalability but can be limited in conductivity and washability [13]. At the same time, 3D knitting and additive manufacturing provide high precision and customisation but may involve complex setups and higher production costs [10]. The choice of method depends on the specific application requirements, such as flexibility, conductivity, and production volume.

Therefore, UW stands out as an optimal method for smart textile fabrication due to its ability to create strong, conductive bonds without the need for adhesives or additional materials [14]. This technique uses high-frequency vibrations to create localised heat, securely bonding conductive materials. Unlike traditional methods, UW ensures high precision and reliability, making it ideal for integrating electronic components into textiles. Additionally, it offers advantages such as rapid processing, energy efficiency, and minimal material wastage [15]. UW has emerged as a promising technique for fabricating smart textiles, offering numerous advantages. However, several challenges exist in applying this method, which has sparked current research efforts to address these issues.

One primary challenge is achieving strong and reliable bonds between conductive elements and textile substrates. While UW can provide secure connections, ensuring consistent conductivity and durability across various textile materials and conductive elements is non-trivial [16]. Additionally, selecting suitable welding parameters, such as amplitude, pressure, and time, can be challenging. These parameters greatly affect the quality of welds, but their optimal values may vary based on the specific materials and design of the smart textile [17,18,19,20]. Another significant challenge is managing the localised heat generated during the UW process. Excessive heat can damage sensitive electronic components or the textile substrate itself, reducing the functionality and longevity of the smart textile. Moreover, ensuring the uniformity of the welds across large textile surfaces is another pressing challenge, as non-uniformity can lead to weak spots and reduced performance [21].

Furthermore, achieving flexibility and stretchability in smart textiles while utilising UW remains an ongoing research endeavour. Smart textiles are intended to conform to the body or textile structure while retaining functionality. Balancing the demands of strong, conductive bonds with the need for textile flexibility and comfort presents a challenge and material optimisation problem [22,23].

The optimisation of UW parameters plays a vital role in achieving high-quality products. It can be optimised through a comprehensive experimental approach, where pressure and time are systematically tested and analysed to determine the optimal settings for achieving strong and reliable welds. Backpropagation neural network (BPNN) modelling is a highly effective method used in artificial intelligence and machine learning for optimisation and other objectives [24]. With that approach, one can develop artificial neural networks whose internal parameters are optimised sequentially by minimising prediction errors. It starts with an initial model, and then through a process called backpropagation, the error is calculated and passed backwards; thus, the network can learn itself as a more complex pattern and predict from the new data [25]. Various fields, such as biomaterials and manufacturing, have also employed this modelling method [26,27]. This can present the importance of BPNN as an integral element of most modern machine learning schemes.

From the preceding narratives, it can be concluded that UW offers significant promise for smart textile fabrication, but several challenges persist in ensuring consistent performance, reliability, and comfort. Research efforts are directed toward optimising welding parameters, developing high-strength wire-fabric connections, and addressing materials and design challenges. As these challenges are addressed, the potential of smart textiles will be further unlocked for diverse applications. Therefore, the current investigation attempts to fix some previously mentioned barriers concerning optimising the UW parameters to improve the smart fabrics’ strength and electrical characteristics.

## 2. Methodology

Firstly, FEA modelling using the ABAQUS 6.14 software package (Dassault Systèmes Simulia Corp, Johnston, Rhode Island) has been employed to design an ultrasonic piezoelectric transducer, which acts as the heartbeat of the UW system. The FEA tool enabled the simulation and optimisation of the transducer’s geometry to ensure efficient and precise ultrasonic energy generation. Subsequently, the design was fabricated by carefully selecting materials that aligned closely with the FEA model. The transducer was designed to generate consistent ultrasonic vibrations for the experimental work within the study.

After designing the ultrasonic transducer, the effort moved to designing and fabricating a specialised UW setup. This setup was tailored to the unique requirements of joining copper wires with non-woven PVC textiles. During the design phase, components such as the transducer, horn, and fixture were thoughtfully selected and strategically configured to ensure compatibility with the target materials. Furthermore, the setup was accurately constructed to allow for precise control of critical welding parameters, including copper wire diameter in mm, welding time in sec and pressure in bar. Three levels were specified for each of the three UW parameters as shown in Table 1. This precision became instrumental in achieving consistent weld quality and laid the groundwork for the subsequent comprehensive investigation into the effects of welding process parameters.

In order to systematically investigate the sophisticated relationship between welding process parameters and process outcomes, the Design of Experiment (DOE) technique was utilised to conduct number of experiments using Minitab 16 software (Minitab, LLC, Lock Haven, PA, US). The full factorial DOE model comprised 3 parameters with 3 levels for each. A complete combination array consisting of 3^3^ = 27 trials was executed, and the weldability performance was recorded, as showcased in Table 2. This method proved efficient in exploring various parameter combinations while minimising the required experimental runs. The research aimed to collect robust data that could be statistically analysed for patterns and trends by varying welding parameters such as time, pressure, and copper wire diameter.

The experimental data obtained from the 27-array design became the foundation for the next phase. These data were employed to develop an interactive model using the backpropagation neural network (BPNN) technique. The BPNN model was trained on the experimental outcomes, transforming it into a predictive tool for process control and optimisation. Its capacity for learning and generalisation allowed it to offer accurate predictions regarding the relationships between welding parameters and critical factors, such as the heat generated (in terms of welding temperature), joint strength (in terms of peeling force), and post-welding electrical properties (in terms of electrical resistance) of the copper wires. This methodology, from transducer design to BPNN modelling, forged a path toward a comprehensive understanding of the UW process, with significant implications for smart textile applications.

## 3. Transducer FEA Modelling and Manufacturing

**Figure 1:**
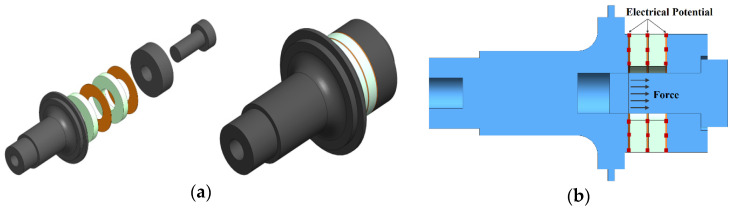
Transducer model (**a**) geometry and (**b**) boundary conditions.

The first step in constructing the FEA model was to build the geometry of the transducer parts. Dimensions of each part of the transducer were calculated based on the acoustic wave equation [28]. After that, the ABAQUS package has been used to build the model geometry shown in Figure 1a. Initially, the front mass geometry was assumed to be stepped. The stepped horn was distinguished by the high amplification of vibration amplitude [28].

Based on the literature, proper materials have been assigned to each transducer’s parts. Aluminium has been used for the front and the back masses. Simultaneously, copper and piezoelectric materials have been allocated for the electrodes and the rings, respectively.

Table 3 presents material properties for defining the materials in the FEA model. Afterwards, loads and boundary conditions are assigned. The pre-stressed bolt load, estimated based on the recommended piezoelectric stack compression state (around 30 MPa), is applied at the bolt cross-section between the back mass and the piezoelectric stack. Boundary conditions (BC) are also used for piezoelectric elements, facilitating electrical potential propagation from the base state [29,30]. Figure 1b illustrates these load and boundary conditions. 

Regarding the FEA model meshing process, the structure meshing technique is preferred for such an electromechanical system [29,30]. Moreover, suitable elements for the different parts of the transducer were selected. All metallic parts were 3D stress quadratic elements (C3D20R), while the piezoelectric rings were a piezoelectric quadratic element (C3D20RE).

**Figure 2:**
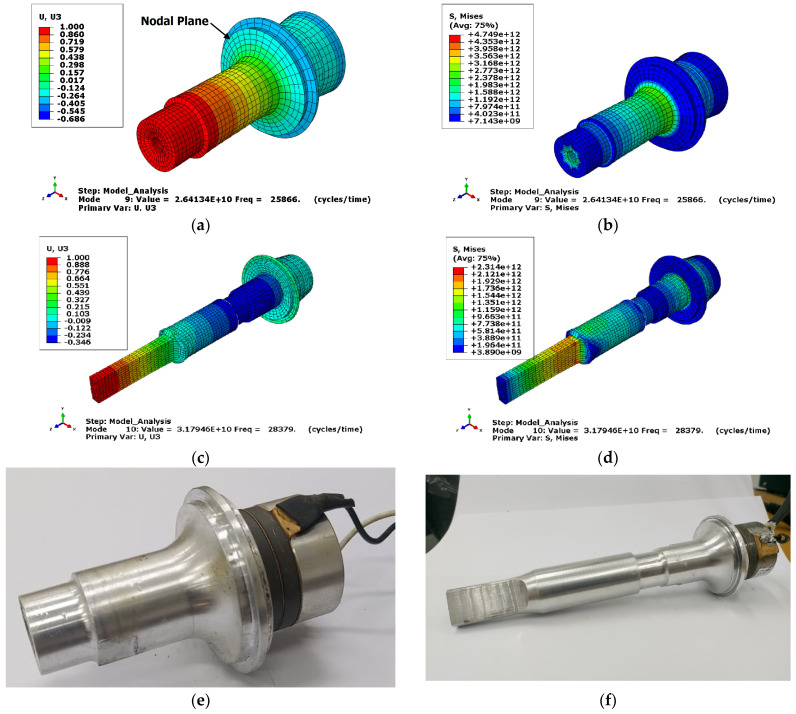
Along the axial direction of the transducer are the (**a**) normalised displacement, (**b**) von Mises stress in MPa, (**c**) normalised displacement transducer with ultrasonic horn, (**d**) von Mises stress in MPa transducer with ultrasonic horn, (**e**) fabricated transducer, and (**f**) fabricated transducer with ultrasonic horn.

Concerning the FEA results, Figure 2a,b illustrates displacement distribution and von Mises stress along the transducer’s axial direction for the longitudinal mode shape observed at 25.87 kHz. The nodal plane position, which supports the transducer during experiments, is also presented. A welding horn with a calculated length was attached to the working end to maximise output amplitude. It should be noted that changes in the transducer mass can influence the system’s resonance frequency. 

Figure 2c,d shows displacement distribution, and von Mises stress along the transducer’s axial direction for the longitudinal mode shape after adding the welding horn. It occurred at 28.38 kHz.

## 4. UW Machine and Experimental Process Design

In the current study, DOE is used to model and analyse the effect of the UW regime on the heat generation, joint strength, and electrical properties of welded joints for smart textiles. The general functional relationship can be written as in Equation (1):

(1) Y=f(D,P,t)

where *Y* represents the dependent variable, while *D*, *P*, and t represent the independent variables of copper wire diameter, welding pressure, and welding time, respectively. The general functional relationship forms the basis of experimental design, defining factors and interactions. Analysis of Variance (ANOVA) assesses their significance once data are collected. Polynomial models, including linear and non-linear components, can be part of this relationship. This allows for a more flexible and accurate representation of the data, capturing potential curvature or interaction effects that may exist. ANOVA is crucial for evaluating these models and identifying statistically significant terms. Together, these tools enable systematic exploration and optimisation. The general form of the ANOVA model with polynomial terms can be represented in Equation (2):

(2) $$Y=\beta_{0}+\beta_{1}X+\beta_{2}X^{2}+\beta_{3}X^{3}+{\ldots +\beta }_{n}X^{n}+\varepsilon$$

In this Equation, *X* represents the independent variable, *β*_0_, *β*_1_, *β*_2_, …, *β_n_* are the coefficients associated with each polynomial term, *X*^2^, *X*^3^, …, *X^n^* represent the different powers of the independent variable, and *ε* defines the error term.

**Figure 3:**
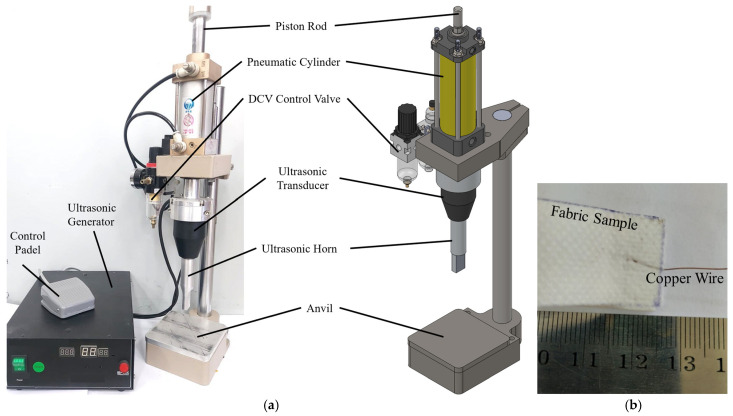
(**a**) Design and fabrication of UW machine; (**b**) produced ultrasonic welded sample for smart textile.

A prototype vertical UW machine was designed to facilitate the experimental process. The UW parameters encompass a maximum power rating of 1 kW and a frequency of 28 kHz. To achieve the desired welding of fabric–copper connections, ultrasonic waves are applied perpendicularly to the surface, transversely engaging with the fabrics. The UW system comprises essential components, including a power supply, a converter, a booster, and a horn (Figure 3a).

This system transforms the electrical energy, supplied at a frequency of 50–60 Hz by the power supply, into mechanical vibration energy oscillating at 28 kHz through the converter. The resulting frictional heat generated by the vibration is then conveyed to the junction surface via the horn, facilitating the formation of a bond between the fabric and copper wire. The welding and holding time can be precisely adjusted within 1.0 s to 10 s, while the pneumatic pressure applied to the welding area can reach up to 1 MPa.

To make the proper contact point between the fabric and the copper wires, non-woven fabrics were stripped to about 60 mm in length and 20 mm in width. The polyester fabric was provided by Kar-tex (Cairo, Egypt) as a material for facemasks, personal protective clothes, diapers, and medical products in the market. Then, plain annealed stranded copper wires (99.97% pure) supplied by Elsewedy Electric (New Cairo, Egypt) with different diameters (0.2, 0.3, and 0.4 mm) were inserted, centred, and integrated between the two pieces of fabric (between 0.05 and 0.08 mm thick each) using an UW machine; the welded sample is shown in Figure 3b.

**Figure 4:**
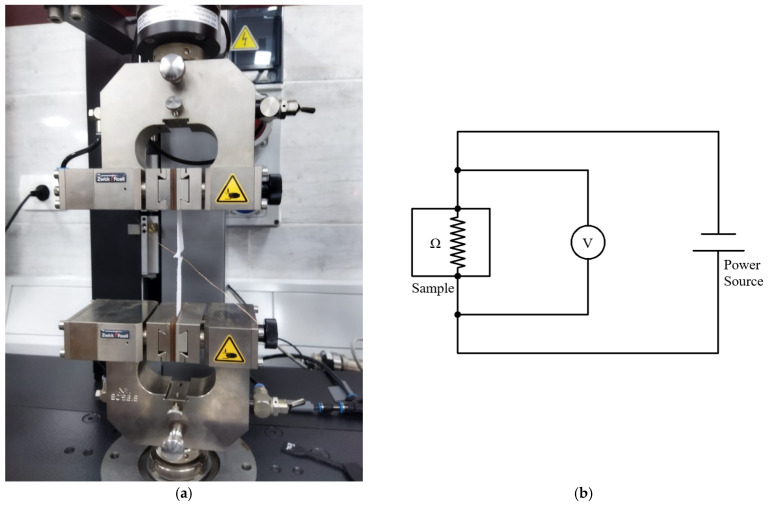
(**a**) Peeling test experimental configuration; (**b**) schematic of electrical resistance measurement setup.

After UW, a Scanning Electron Microscope (SEM) model (SU-70), manufactured by Hitachi High-Tech, Japan, was used to investigate the bonding morphology of the fabric-copper joint. A peeling test was conducted to determine the mechanical strength of the joint to analyse the adhesion of electronic interconnectors. Therefore, the self-developed peeling test is ideal for inspecting joints between copper wires and textiles. The sample was clamped and fixed on the grips of the Zwick tensile testing machine model (Z010) manufactured by Zwick\Roell, Germany, with a 10 KN maximum capacity. The force was applied to the textile with a 10 mm/min travel speed. The configuration of the peeling test is illustrated in Figure 4a.

A four-wire measuring instrument is used to measure the contact electrical resistance, as illustrated in Figure 4b. Two of the four contacts provided a current of 1 A. A voltmeter over the two remaining conductors measured the falling voltage at the resistor. Based on Ohm’s law, it was possible to calculate the electrical resistance of the contact. The contact electrical resistances were in the range of only a few milliohms. Finally, an infra gun was used to measure the temperature through welding time.

## 5. Development of Feed Forward Back Propagation Network (FFBPN) for Prediction of Smart Textile Connection Quality

An artificial neural network (ANN) is a computational tool inspired by the complex neural networks of the human brain. It is excellently tuned to discern intricate patterns, make data-driven predictions, and quickly resolve complex engineering challenges. ANNs are adept at accurate outcome prediction and find versatile applications in various engineering domains. ANNs, as computational models, require training to learn and make predictions or classifications. The training process involves adjusting the neural network’s internal parameters (weights and biases) based on a dataset to minimise prediction errors.

MATLAB R2020a was used to develop the FFBPN model. The FFBPN model was developed by taking the three parameters (wire diameter, pressure, and time) from the data and considering them as inputs. Heat generation, joint strength, and electrical properties were then taken one at a time as outputs. Three phases of model development were conducted, using 70%, 15%, and 15% of the available data for training, testing, and validation, respectively. If the model meets the performance criteria established during the training stage, it is considered successfully developed and validated; then, the model proceeds to the testing stage. Otherwise, it is recalled for retraining in the first stage. It is deemed validated once the model passes the testing stage and meets the performance criteria. The following four steps comprise the backpropagation method’s training algorithm: initialisation of weights, feed-forward, backpropagation of errors, and updating the weights and biases, respectively. The proposed network architecture involves three input neurons for each input parameter, an output layer with one neuron corresponding to one output at a time, and a single hidden layer of neurons.

The Levenberg–Marquardt (LM) backpropagation algorithm will be used if the model does not meet the expected values [31]. This allows the procedure to be repeated until it finds the optimal requirement. When determining the architecture of a backpropagation neural network (BPNN) using a trial-and-error method, several design criteria can guide the process illustrated in Table 4.

**Figure 5:**
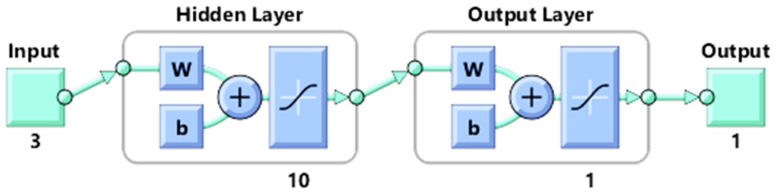
Proposed network architecture. The colours represent different types of layers or components. Turquoise colour represents input/output data and blue colour represents components of each layer and its functions like summation and transfer functions. Arrows depict the flow of in-formation, indicating how data moves from one layer to another. The plus symbol could be used to denote the addition of bias and weights in the equations that govern the calculations within the network.

After the input layer receives the signals from the other source, the hidden layer converts them into a form that the output signal can utilise. This study’s suggested neural network architecture is 3-10-1, as seen in Figure 5.

In the current investigation, experimental data from the DOE emphasises the development of an interactive backpropagation neural network (BPNN) model. This BPNN model was trained on experimental data to provide a predictive tool for process control and optimisation.

## 6. Results and Discussion

### 6.1. Preliminary Results of the UW Process

**Figure 6:**
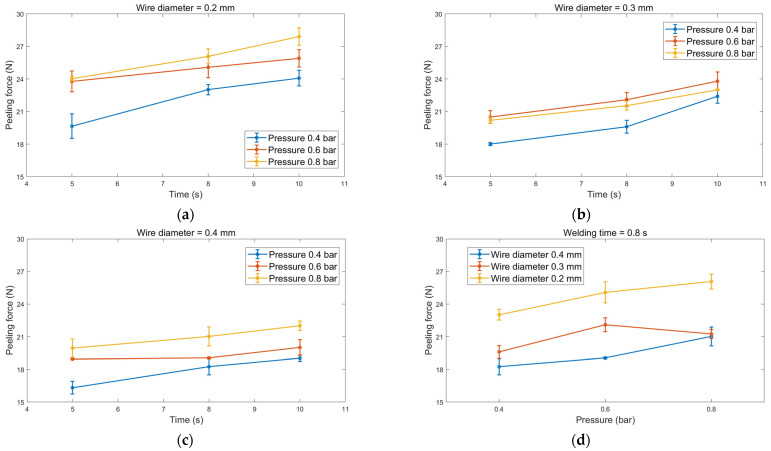
The peeling force for different welding pressures and welding time dependencies for wire diameter of (**a**) 0.2 mm, (**b**) 0.3 mm, (**c**) 0.4 mm, and (**d**) peeling force for different pressures.

The mechanical peeling tests showed a significant relationship between welding parameters and joint strength, as illustrated in Figure 6. For instance, with a 0.2 mm diameter copper wire at 0.4 bar pressure, the peeling force increased from 19.6 N to 24 N as welding time extended from 5 s to 10 s. This trend was consistent across different pressure levels, with the highest peeling forces of 26 N and 28 N observed at 0.8 bar pressure for welding times of 8 s and 10 s. Higher pressure levels enhanced bonding between the non-woven fabric layers by promoting intermolecular contact and improving adhesion strength.

The positive correlation between peeling force and welding time underscored that longer welding times facilitate thorough bonding and material interdiffusion, increasing joint strength. Conversely, increasing copper wire diameter led to a decrease in peeling force. This phenomenon was attributed to the wire’s heat dissipation characteristics. A larger wire diameter resulted in more efficient heat dissipation, weakening the bonding between fibres due to reduced heat input and diffusion. These findings emphasise the intricate interplay between welding parameters and joint strength in UW of non-woven fabric. While both pressure and time are vital, pressure appeared to have a more pronounced impact.

The electrical resistance measurements conducted on the wire joints highlighted the significant influence of welding parameters on electrical resistance. The electrical resistance values exhibited variations dependent on both wire diameter and welding conditions. Specifically, for wire diameters of 0.2, 0.3, and 0.4 mm, the recorded electrical resistance values were 0.02, 0.04, and 0.06 Ohm, respectively. All measurements were taken on wires with a fixed length of 60 mm to ensure equitable comparisons.

**Figure 7:**
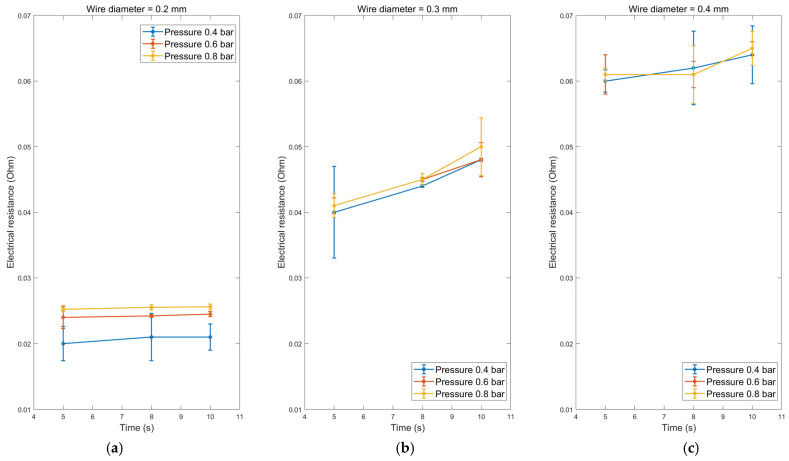
The electrical resistance for different welding pressures and welding time dependencies for wire diameter of (**a**) 0.2 mm, (**b**) 0.3 mm, and (**c**) 0.4 mm.

The electrical resistance data show a slight increase with welding time. For instance, with a 0.2 mm wire diameter at 0.4 bar pressure, electrical resistance increased from 0.02 to 0.021 Ohm, as depicted in Figure 7. However, a more pronounced rise in electrical resistance occurred with heightened pressure. At 0.8 bar pressure and a welding duration of 10 s, electrical resistance reached 0.0256 Ohm. Similar trends materialised with 0.3 and 0.4 mm wire diameters.

This relationship between wire diameter and electrical resistance can be attributed to the alterations in cross-sectional area, which led to heightened electrical resistance. While pressure could potentially deform the wire and influence its cross-section, the predominant factor affecting electrical resistance was deemed to be the cross-sectional area. These findings underscore the imperative consideration of welding parameters and wire diameter concerning electrical resistance measurements. Minimising electrical resistance in welded joints is critical to avoid electrical losses and inefficiencies.

The observed correlation between wire diameter and electrical resistance can be attributed to alterations in the weldment area, resulting in elevated electrical resistance. The increase in electrical resistance is primarily attributed to various defects induced by high welding pressure as revealed by SEM observation. As expected, the presence of unbonded regions at the joint interface contributes to an increase in electrical resistance. Consequently, during charging cycles, the elevated temperature intensifies the electrical resistance, leading to overall performance degradation. Furthermore, the diffusion rate of elements appears to be influenced by welding pressure and time. Previous studies have frequently highlighted that the excess concentration of vacancies generated through severe plastic deformation enhances diffusion around the interface during ultrasonic welding [32,33]. As a result, faster diffusion at the interface with minimal pressure is preferable for achieving a conductive joint. This interpretation aligns with the observed electrical resistance of the 0.4 mm wire as both time and pressure increase, as shown in Figure 6c. These findings underscore the critical importance of considering welding parameters and wire diameter when conducting electrical resistance measurements. Minimising the electrical resistance of welded joints is imperative to mitigate electrical losses and enhance overall efficiency.

**Figure 8:**
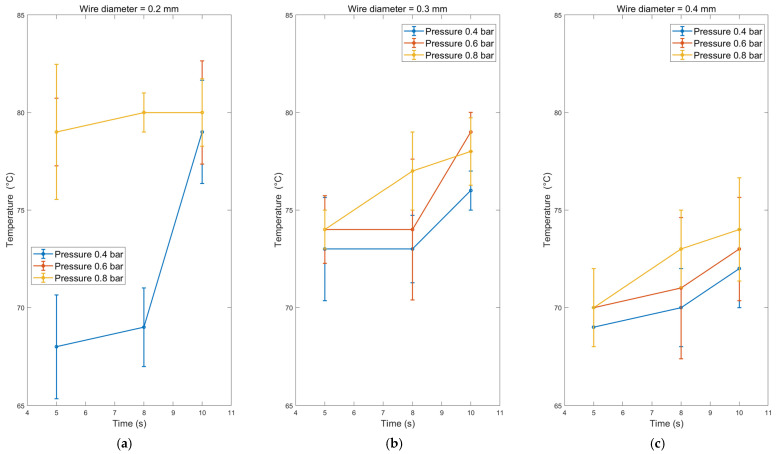
The temperature for different welding pressures and welding time-dependent for wire diameter (**a**) 0.2 mm, (**b**) 0.3 mm, and (**c**) 0.4 mm.

The temperature measurements during the UW process for a 0.2 mm copper wire revealed a consistent trend across different time durations and pressures. At a pressure of 0.4 bar, the temperature increased from 68 °C to 79 °C as the welding time increased from 5 s to 10 s, as shown in Figure 8a. This trend was observed consistently across all tested pressures. The highest temperature recorded was 80 °C at a pressure of 0.8 bar and a welding time of 10 s.

When considering the effect of wire diameter on temperature, it was observed that the temperature slightly decreased as the wire diameter increased. The minimum temperature and the highest pressure were recorded at a welding time of 5 s. This observation suggests that temperature is primarily influenced by the wire diameter, with larger diameters dissipating welding heat more rapidly Figure 8. Furthermore, the effects of welding time and pressure increased the temperature for each independent wire diameter. This indicates that both time and pressure contribute to generating heat during the welding process.

The observed temperature trends can be attributed to the thermal characteristics of the copper wire and the energy input during UW. As the welding time increases, more energy is delivered to the wire, resulting in a higher temperature. Similarly, increased pressure can increase temperature due to enhanced energy transfer. However, the effect of wire diameter on temperature is linked to its ability to dissipate heat efficiently. These findings emphasise optimising welding parameters to achieve the desired temperature range for effective and reliable bonding. The results suggest that shorter welding times and lower pressures may help reduce excessive temperature generation, mainly when working with smaller wire diameters. Additionally, careful consideration of the wire diameter is necessary to ensure proper heat dissipation and prevent overheating.

**Figure 9:**
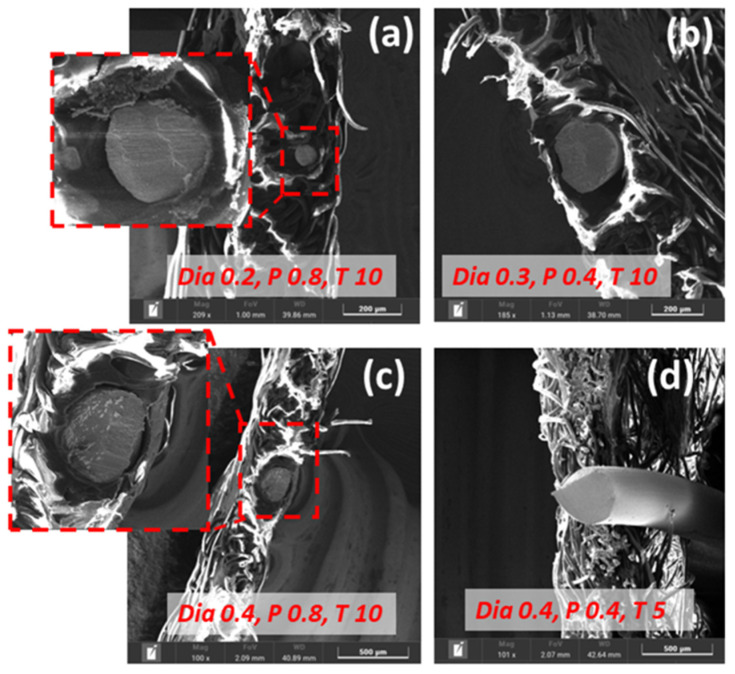
SEM of fabric–copper UW under different parameters. (**a**) Wire diameter 0.2, Pressure 0.8 bar & Time 10 s. (**b**) Wire diameter 0.3, Pressure 0.4 bar & Time 10 s. (**c**) Wire diameter 0.4, Pressure 0.8 bar & Time 10 s. (**d**) Wire diameter 0.4, Pressure 0.4 bar & Time 5 s.

Figure 9 shows an SEM of the welded joint under different parameters. The 0.4 mm copper wire with the highest pressure and time appeared to have an efficient bonding interface morphology. With increased welding time and fixing other parameters, the wire was squeezed into the gap regions of the fabrics. The high-frequency vibration caused friction and subsequent heat, which softened the copper in the bonding. It was then squeezed into the arc-like movement path. However, if the time was short, the energy and temperature produced from friction would not be sufficient, and material plastic flow became more difficult even if the pressure continued to increase. In addition, the wire diameter played a vital role in transferring the heat produced by friction, as indicated by Figure 9a,c.

As per UW analysis, after ultrasonic vibration was activated, the horn directly contacted the fabric layers, squeezed and rubbed it, and a relative sliding motion occurred, generating heat and bonding through the acoustic softening effect. Firstly, a virtual bond is formed quickly between the layer’s interfaces. With the increase in welding time, the virtual bond would tear firstly and join again under the action of the horn pressure and shear force, and then produce bonding, interlocking to form a robust bond region, which leads to the increase in the joint bonding strength. However, since the force and relative sliding required to create a bond between the fabric layers need to be transferred through the copper wire, there is a certain hysteresis and attenuation, and this makes the time increase not always the influential parameter (Figure 9c,d).

### 6.2. Statistical Modelling of the UW Process Using the Design of the Experiment (DOE)

Regarding the DOE results, for the current investigation, a 95% level of statistical significance was selected. The determined correlation coefficients R^2^ are 80.5%, 95.3%, and 99.37% for heat generation, joint strength, and electrical properties of the welded joints, respectively, showing that the data match the models very well and can be used to derive it. The models that resulted from the analysis can be expressed as:

(3) $$T=40.7+81.1\;D+68.0\;P+0.11\;t-105.6\;D^{2}-26.4\;P^{2}+0.122\;t^{2}-50.0\;D\;P-2.11\;D\;t-1.23\;P\;t$$

(4) $$S=14.57-47.8\;D+33.5\;P+0.886\;t+65.9\;D^{2}-17.06\;P^{2}+0.0314\;t^{2}-7.8\;D\;P-\;1.577\;D\;t-0.482\;P\;t$$

(5) Ω=−0.02659+0.2019 D+0.01666 P+0.000229 t−0.0096 D P−0.00030 D t +0.000024 P t

where *D*, *P*, and *T* represent the independent variables of copper wire diameter, welding pressure, and welding temperature, where t, S, and Ω represent welding time, peeling force, and electrical resistance, respectively.

The impact of UW parameters on heat generation, joint strength, and electrical properties are tested experimentally for 3^3^ = 27 combinations involving three variables, and a statistical approach is used to clarify the relative effects of different process parameters on the responses. Thus, a three-factor Analysis of Variance (ANOVA) approach is applied to the collected dataset using the Minitab program to examine the effects of various factors, including wire diameter, pressure, and time on the produced joints. ANOVA analysis results are displayed in Table 5 with a confidence level of 0.95. In this study, the degrees of freedom for single factors were 2, the degrees of freedom for interaction between factors were 4, and the error number was 8.

For temperature, the wire diameter and pressure significantly influence the experimental results when their contributions to the response are 38.11% and 27.58%, respectively. Meanwhile, the interaction between factors has no significant influence on temperature. The wire diameter, welding time, and pressure are the significant single factors that affect peeling force. The interaction between wire diameter and pressure follows it. According to the results of ANOVA, wire diameter and pressure are significant single factors followed by interaction between wire diameter and pressure that affect electrical properties.

**Figure 10:**
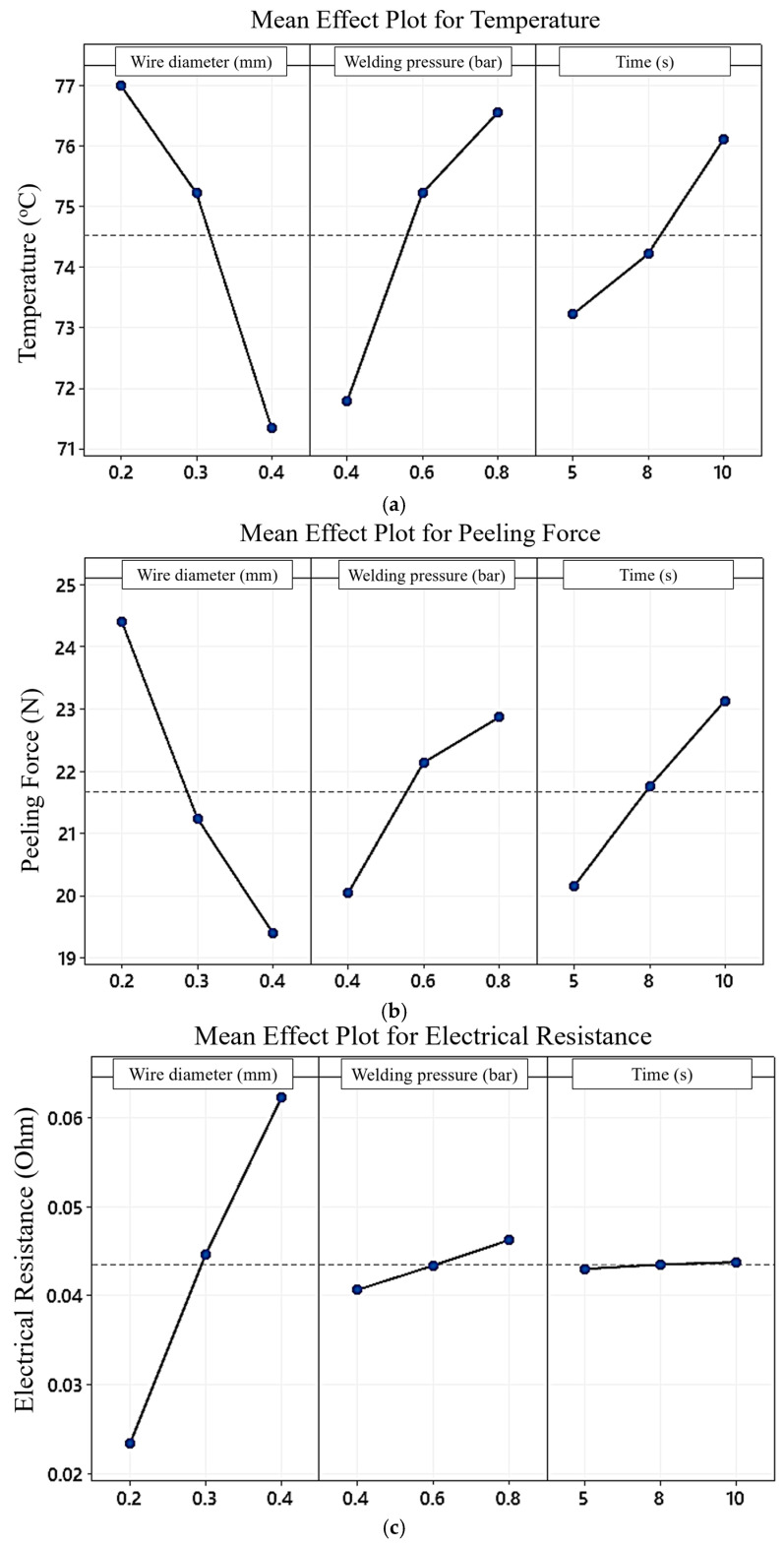
Mean effect plot of (**a**) temperature, (**b**) joint strength, and (**c**) electrical resistance.

The main effect plots show how each factor affects the response characteristic. As shown in Figure 10a,b, each temperature and peeling force trend decreased by increasing the diameter and decreasing pressure and time. However, the electrical resistance of weldment increased by increasing each diameter and pressure, as shown in Figure 10c. Meanwhile, the change in levels of time from 5 s to 10 s has a slight, almost negligible impact on electrical resistance.

Subplots (a), (b), and (c) show a preponderance of the effect of a diameter of 0.4 mm. From (a) and (b), it is concluded that the optimum combination of each process parameter for lower heat generation and joint strength is meeting at wire diameter, 0.4 mm; pressure, 0.4 bar; and time, 10 s. Figure 10c shows that the optimum combination of each process parameter for higher electrical resistance is meeting at wire diameter, 0.4 mm; pressure, 0.8 bar; and time, 5–10 s; this has the same effect.

### 6.3. Comparison of Experiment, BPNN and FFD Results

**Figure 11:**
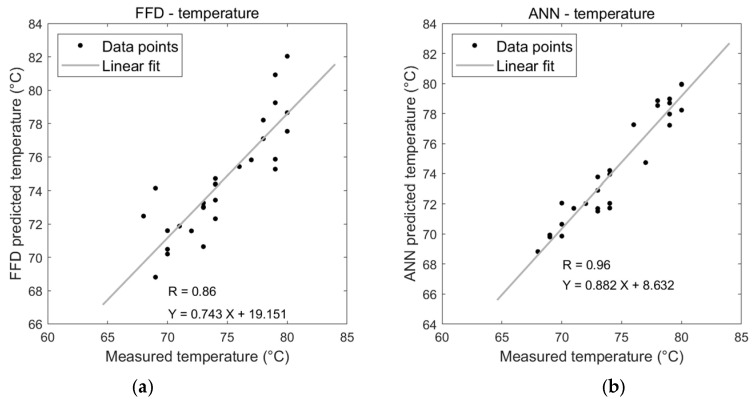

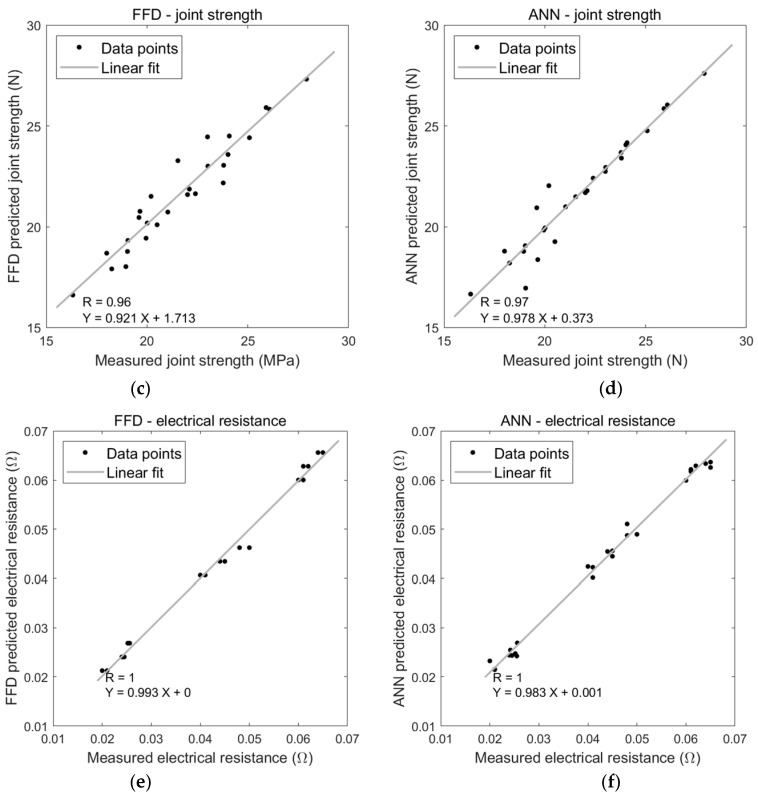
Graphical comparison of correlation plots for FFD and ANN Models for weldment proprieties. (**a**) Correlation plot of FFD for temperature. (**b**) Correlation plot of ANN for temperature. (**c**) Correlation plot of FFD for peeling force. (**d**) Correlation plot of ANN for peeling force. (**e**) Correlation plot of FFD for electrical resistance. (**f**) Correlation plot of ANN for electrical resistance.

A comparison was conducted with experiment results to evaluate the predictive performance of BPNN and FFD. The performances of developed models via FFD and ANN of temperature, mechanical peeling force, and electrical resistance of the welded joints were evaluated using correlation coefficient (R) as shown in Figure 11a–f. Figure 11a,b show the correlation coefficient diagrams among predicted values of each approach and the observed values for measured temperature; the R of the FFD model equals 0.862, while the R of the ANN model equals 0.955. The correlation value for the overall performance of ANN for peeling force is very close to that of the FFD model, which equals 0.967 and 0.96, respectively, as shown in Figure 11c,d. The correlation coefficient R for both models resulting from both methods, FFD and ANN, was approximately the same and very close to 1 when studying electrical resistance, which is equal to 0.997, as shown in Figure 11e,f.

ANN has been found to perform better than FFD or other techniques for non-linear systems. Meanwhile, many repeated calculations are required because of the resilience of the ANN model. This technique also obscures the input factors’ contributions and how they interact. The performances of FFD and BPNN models are tested by statistical criteria such as the coefficient of correlation (R), which provides the value of proportionality between the data from the experiment and the estimation, while the accuracy of the estimation results is measured by mean absolute percentage error (MAPE) which can be calculated by following the formula, The results are summarised in Table 6.

(6) $$MAPE=\frac{1}{n}\sum_{i=1}^{n}\left|\frac{y_{a,i}-y_{p.i}}{y_{a.i}}\right|$$

where *n* is the number of experiments, $y_{a,i}$ is the actual value, and $y_{p,i}$ is the predicted value.

Finally, according to the developed FFD and ANN models, the percentage of overall error for ANN and FFD for each heat generation, joint strength, and electrical properties is approximately 3.91%, 6.84%, and 0.49%, respectively, confirming the high accuracy of the predictive model. The FFD model results in an average error percentage of 4.69% and 0.7% for joint strength and electrical properties, respectively, while the rate of overall error of modelling heat generation is 19.52%, which means there may be other variables affecting the process that have not yet been taken into account which are responsible for this amount of variation. The results indicate that both the developed models are highly accurate in predicting the joint strength and electrical properties; however, ANN is more accurate than FFD in predicting heat generation model. The analysis shows that wire diameter mainly contributes 38.11%, 55.76%, and 97.59% to heat generation, joint strength, and electrical properties, respectively. Although the diameter of the wire is considered the primary variable affecting all output responses, its relatively small contribution rate of 38.11% in heat generation compared to the other responses, reflects that the levels used in this experiment were very narrow and reflected in that percentage.

## 7. Conclusions

This work involves the design of a piezoelectric transducer in parallel with the prototype development of a UW machine. This integrated approach aims to explore the impact of welding parameters, such as time, pressure, and wire diameter, on the joint efficiency of smart textiles. The experimental results derived from this endeavour were subsequently employed in the optimisation process, combining full factorial design (FFD) and artificial neural networks (ANNs) to enhance and control UW processes. The main insights can be summed up as follows:

Both pressure and time are vital; pressure appeared to have a more pronounced impact. The results suggest that an optimal combination of pressure and time can be determined to achieve the desired joint strength, considering specific wire diameter and material characteristics.

Reducing wire diameter and controlling pressure becomes instrumental in achieving lower electrical resistance values and enhancing the overall electrical properties of the joints.

According to experimental research, the minimum heat generation, maximum strength, and maximum electrical resistance of UW of copper wire and non-woven PVC textiles can reach 68 °C, 27.91 N, and 0.065 Ohm. The wire diameter significantly impacts the joint strength, electrical properties, and heat generation followed by welding pressure, but the welding time makes a slightly significant difference. Given the interactions between factors, the interactions between wire diameter and welding pressure slightly affect FFEAct responses. However, the interaction between the other two groups has no significant impact on the experimental results.

The proposed FFD and ANN models accurately predict the UW process’s heat generation, joint strength, and electrical properties to join copper wires with non-woven PVC textiles. However, the predictive capability of the FFD model shows a high error percentage of 19.52% only in the case of heat generation. This reflects the necessity of reconsidering the size of the orthogonal array, which plays a critical role in the efficiency of the predictive model.

The ANN with a 3-10-1 architecture is an optimum network with a high correlation coefficient obtained on validation datasets. The model accuracy of ANN was found to be better than FFD, and the former was found to be statistically robust and accurate in predicting heat generation. At the same time, they are almost identical in robustness and accuracy in predicting joint strength and electrical properties.

## Figures and Tables

**Table 1:** UW parameters.

Parameters	Levels		
	−1	0	+1
Wire diameter (mm)	0.2	0.3	0.4
Pressure (bar)	0.4	0.6	0.8
Time (s)	5	8	10

**Table 2:** Full 27-parameters array for the main UW input and output responses.

Experiment No.	Input Welding Parameter			Output Response		
	Diameter	Pressure	Time	Heat Generation	Joint Strength	Electrical Properties
	(mm)	(bar)	(s)	(°C)	(N)	(Ohm)
1	0.2	0.4	5	68	19.65	0.02
2	0.2	0.4	8	69	23.02	0.021
3	0.2	0.4	10	79	24.08	0.021
4	0.2	0.6	5	79	23.78	0.024
5	0.2	0.6	8	80	25.08	0.0242
6	0.2	0.6	10	80	25.91	0.0245
7	0.2	0.8	5	79	24.02	0.0252
8	0.2	0.8	8	79	26.08	0.0255
9	0.2	0.8	10	80	27.91	0.0256
10	0.3	0.4	5	73	18	0.04
11	0.3	0.4	8	74	19.6	0.041
12	0.3	0.4	10	74	22.4	0.041
13	0.3	0.6	5	73	20.5	0.044
14	0.3	0.6	8	74	22.1	0.045
15	0.3	0.6	10	77	23.8	0.045
16	0.3	0.8	5	76	20.2	0.048
17	0.3	0.8	8	78	21.53	0.048
18	0.3	0.8	10	78	23	0.05
19	0.4	0.4	5	69	16.32	0.06
20	0.4	0.4	8	70	18.25	0.061
21	0.4	0.4	10	70	19.03	0.061
22	0.4	0.6	5	70	18.95	0.062
23	0.4	0.6	8	71	19.05	0.061
24	0.4	0.6	10	73	20.01	0.061
25	0.4	0.8	5	72	19.95	0.064
26	0.4	0.8	8	73	21.03	0.065
27	0.4	0.8	10	74	22.01	0.065

**Table 3:** Physical properties of transducer component materials.

No.	Material	Young’s Modulus (GPa)	Density (Kg/m^3^)	Poisson’s Ratio	Wave Velocity (m/s)	Characteristic Acoustic Impedance (×10^6^ Ns/m^3^)
1	Aluminium (5083)	70.3	2660	0.33	5140	13.67
2	Steel (AISI 1045)	200	7870	0.3	5040	39.7
3	Piezoelectric (PZT)	73	7700	-	3080	23.72
4	Copper (99.97% pure)	115	8900	0.31	3595	31.9

**Table 4:** The configurations for the BPNN used for the regression problems.

Parameter	Value
Network Type	Feed-forward backpropagation
Number of neurons in the hidden layer	4:35, used value was 10
Number of hidden layers	1:5, used value was 1
Training function	Trainlm
Transfer function	sigmoid function Tansig
Learning Rate	0.05
Performance goal	0.0001

**Table 5:** Analysis of Variance (ANOVA) Test.

	Source	Degrees of Freedom (DF)	Adjusted Sums of Squares (Adj SS)	Adjusted Mean Squares (Adj MS)	F-Value	*p*-Value
Heat generation	Diameter (mm)	2	151.185	75.593	14.93	0.002
	Pressure (bar)	2	109.407	54.704	10.80	0.005
	Time (s)	2	38.741	19.370	3.82	0.068
	Diameter (mm) × Pressure (bar)	4	41.481	10.370	2.05	0.180
	Diameter (mm) × Time (s)	4	8.148	2.037	0.40	0.802
	Pressure (bar) × Time (s)	4	7.259	1.815	0.36	0.832
	Error	8	40.519	5.065		
	Total	26	396.741			
Joint strength	Diameter (mm)	2	114.759	57.3797	341.37	0.000
	Pressure (bar)	2	38.578	19.2892	114.76	0.000
	Time (s)	2	39.914	19.9569	118.73	0.000
	Diameter (mm) × Pressure (bar)	4	5.883	1.4706	8.75	0.005
	Diameter (mm) × Time (s)	4	2.953	0.7383	4.39	0.036
	Pressure (bar) × Time (s)	4	2.361	0.5902	3.51	0.062
	Error	8	1.345	0.1681		
	Total	26	205.793			
Electrical properties	Diameter (mm)	2	0.006787	0.003393	13,278.70	0.000
	Pressure (bar)	2	0.000141	0.000070	275.04	0.000
	Time (s)	2	0.000003	0.000001	5.33	0.034
	Diameter (mm) × Pressure (bar)	4	0.000020	0.000005	19.43	0.000
	Diameter (mm) × Time (s)	4	0.000001	0.000000	0.81	0.551
	Pressure (bar) × Time (s)	4	0.000001	0.000000	1.25	0.364
	Error	8	0.000002	0.000000		
	Total	26	0.006954			

**Table 6:** Comparison of statistical analysis between FFD and ANN models.

	Output					
	Heat Generation		Joint Strength		Electrical Properties	
	FFD	ANN	FFD	ANN	FFD	ANN
R	0.862	0.955	0.96	0.967	0.997	0.997
MAPE	0.018153	0.0078	0.0297	0.021998	0.0257	0.0231

## Data Availability

The data supporting this study’s findings are available on request from the corresponding author.
